# Supplementary material for: LANA-specific CD4+ effector T cells accumulate at the site of KSHV infection in humanized mice
Source: Nat Commun. 2025 Dec 5;17:282. doi: 10.1038/s41467-025-66992-2 (PMC12783722; doi:10.1038/s41467-025-66992-2)
Supplement: Supplementary file 1 — Supplementary Information [file 41467_2025_66992_MOESM1_ESM.pdf]

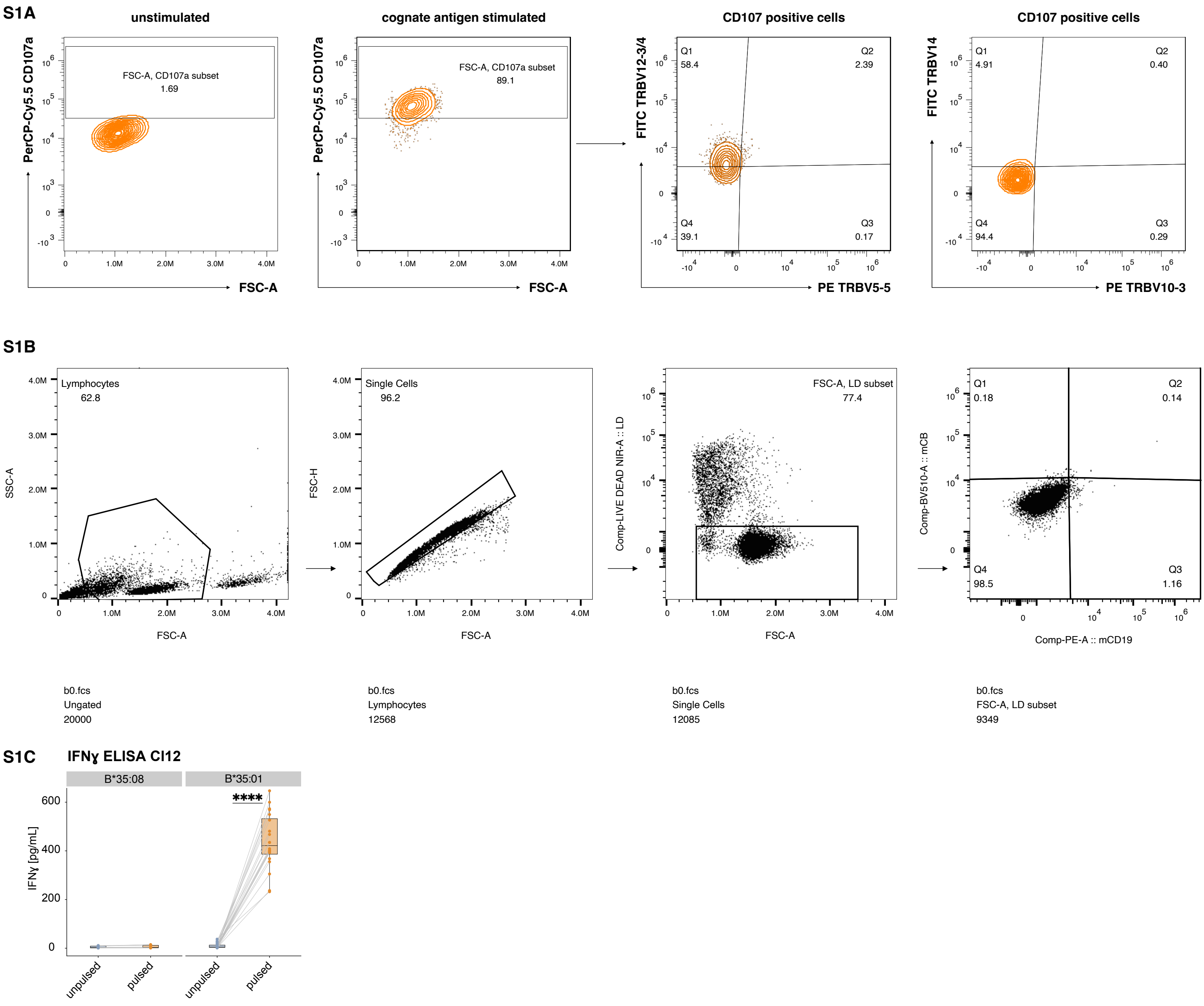

Figure S1 Generation of TCR-transduced T cells: (S1A) Flow cytometry contour plots showing the gating strategy to identify the TCR Vß of the reactive subpopulation. Cognate antigen simulated T cells were gated for CD107a$^+$ cells and further gated for the different TCR Vß. Exemplary plots for 4 different TCR Vß are shown, with positive staining for TRBV12-3/4. **(S1B)** Gating Strategy for transduced and untransduced T cells. Single, live lymphocytes were gated for mCD19 (PE) and mCB (BV510). **(S1C)** IFNɣ ELISA of Cl12 TCR-transduced T cells co-cultured with HLA-B*35:08$^+$ or HLA-B*35:01$^+$ untreated or cognate peptide pulsed LCLs. Graph shows boxplots and individual values of 3 (B*35:08) or 4 (B*35:01) independent experiments, with T cells from 8 (B*35:01) or 6 (B*35:08) different donors, tested against 1 (B*35:08) or 3 (B*35:01) different LCLs. Paired two-tailed t-test. *$p < 0.05$, **$p < 0.01$, ***$p < 0.001$, ****$p < 0.0001$

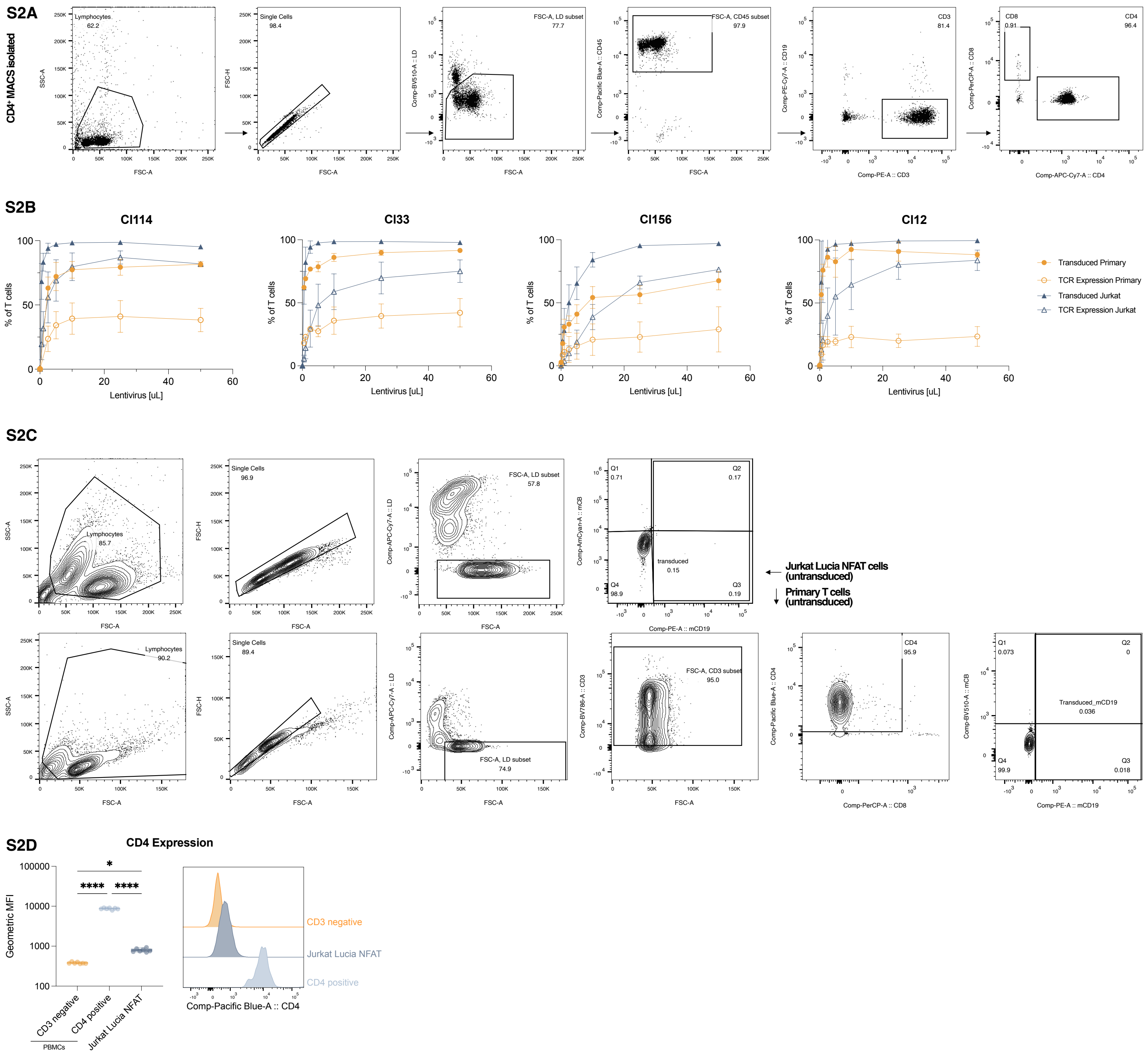

Figure S2 Generation of primary TCR-transgenic T cells: (S2A) Gating Strategy to assess purity of CD4 (exemplary plot) or CD8 MACS isolated primary T cells. Single, live lymphocytes were gated for hCD45+, hCD3+, hCD4+ or hCD8+ T cells. **(S2B)** Frequency of transduced (mCD19+) and TCR expressing (mCB+) T cells (y-axis) as assessed by flow cytometry shown for different amounts of lentivirus added (x-axis). Jurkat cells are shown in blue, primary T cells in orange. Graph shows mean ± SD of n=2 (Cl114 & Cl33 primary, Cl156 Jurkat), n=3 (Cl114 & Cl12 & Cl33 Jurkat), n=4 (Cl12 & Cl156 primary) independent experiments. adjusted p=0.0134 or <0.0001 **(S2C)** Gating strategy showing single, live Jurkat Lucia NFAT cells gated for mCD19+ transduced cells and mCD19+mCB+ TCR expressing cells (top row). Primary T cells were additionally gated for hCD3+, hCD4+ or hCD8+ T cells (bottom row). **(S2D)** Boxplot and individual datapoints of geometric mean fluorescent intensity (MFI, left) and representative flow cytometry histograms (right) of CD4 expression on Jurkat Lucia NFAT cells or primary CD3+CD4+ or CD3- PBMCs. Ordinary one-way ANOVA with Tukey's multiple comparisons test, n=7. *p < 0.05, **p < 0.01, ***p < 0.001, ****p < 0.0001

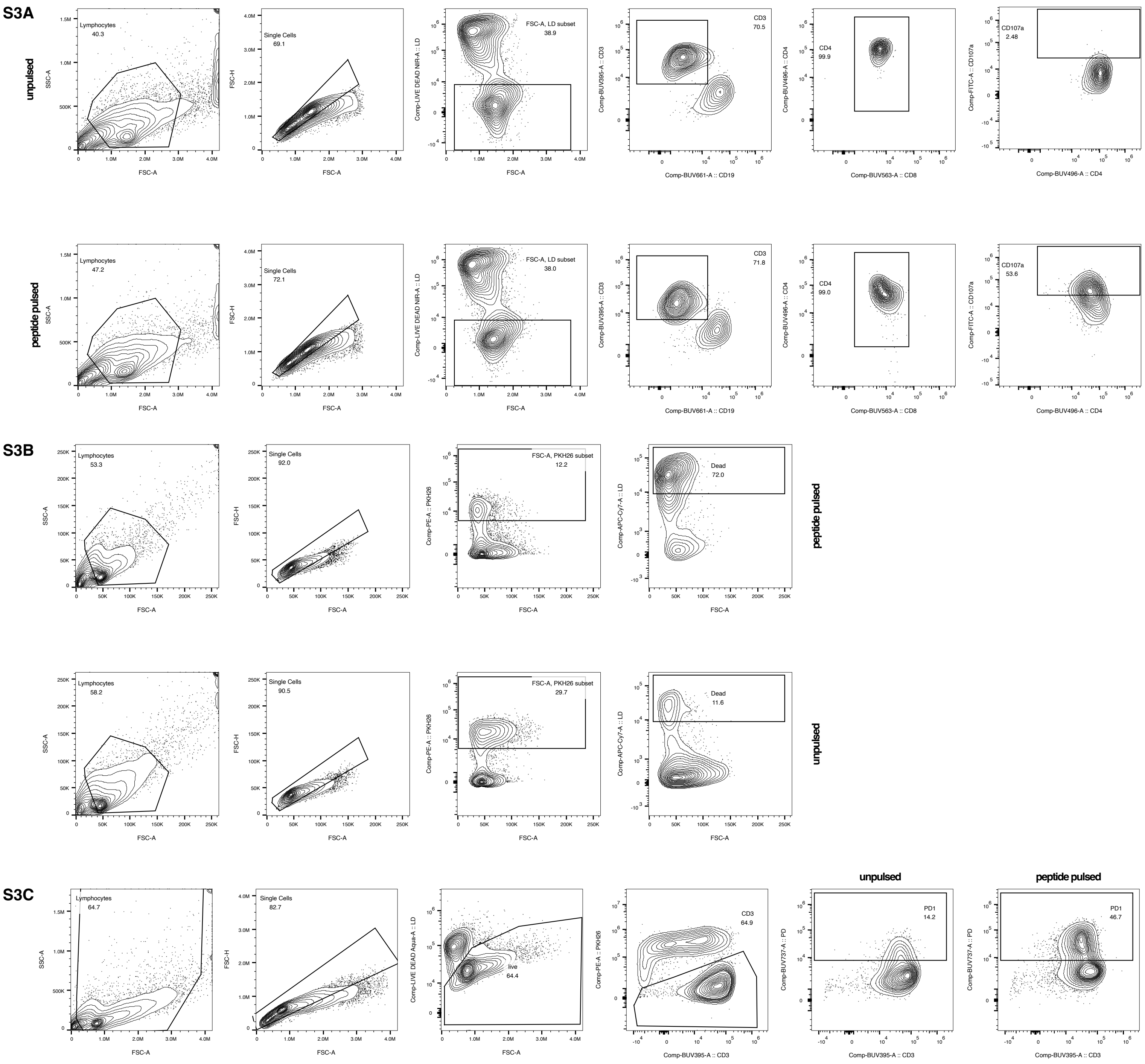

Figure S3 Stimulation of primary TCR-transduced T cells with conate peptide-pulsed target cells: (S3A) Gating strategy to assess the frequency of degranulating or cytotoxic cells. Single, live hCD3$^+$, hCD4$^+$ or hCD8$^+$ T lymphocytes were gated for CD107$^+$ (shown in the exemplary plot) or for the intracellular markers shown in Figure 2F-G. **(S3B)** Gating strategy to assess the frequency of killed target cells. Single, live, PKH26$^+$ target cells were gated for Zombie NIR positive dead cells. **(S3C)** Gating strategy to assess the expression of surface markers indicated in Figure 2F-G. Single, live, PKH26$^-$CD3$^+$ were gated for surface marker expression as shown for PD1 in the exemplary plot.

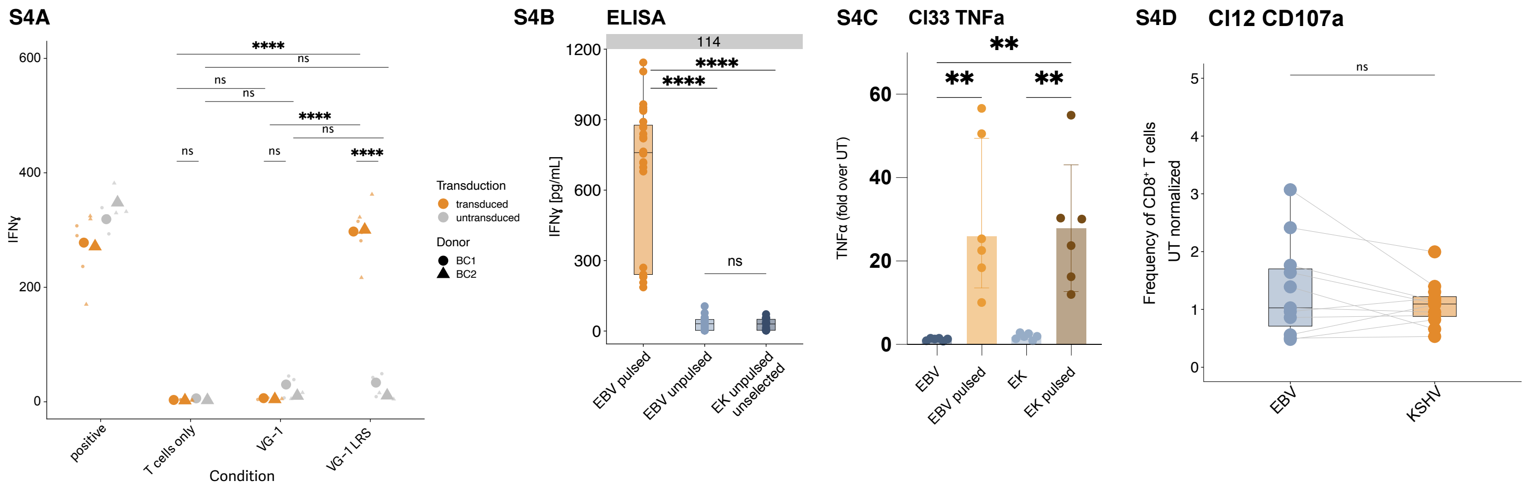

Figure S4 TCR-transduced T cells fail to recognize KSHV-infected PEL cell lines: (S4A) IFNɣ ELISA of Cl33 TCR-transduced or untransduced T cells after overnight co-culture with LRS peptide-pulsed or unpulsed KSHV-infected VG-1 PEL cell lines, with medium only as negative control or with PMA/Ionomycin as positive control. Graph shows mean IFNɣ secretion per T cell donor and transduction (large symbols) of three independent experiments (small symbols). Linear mixed-effects model with fixed effects for condition, transduction and their interaction and random intercept for donor, followed by Holm-adjusted pairwise comparison. Comparisons with positive controls are not shown. **(S4B)** IFNɣ ELISA of Cl114 TCR-transduced T cells co-cultured with HLA-matched untreated (EBV unpulsed) or peptide-pulsed LCLs (EBV pulsed) or with unselected EK LCLs (EK unpulsed unselected). Graph shows boxplots and individual values of 2 independent experiments, with T cells from 2 different donors, tested against 7 different LCLs. Ordinary one-way ANOVA followed by Tukey's multiple comparisons test. **(S4C)** TNF ELISA of Cl33 TCR-transduced T cells co-cultured with HLA-matched untreated or cognate peptide-pulsed EBV-only LCLs or with selected EK LCLs. Graph shows mean ± SD and individual values of one experiment with T cells from 2 different donors, tested against 3 different LCLs. Ordinary one-way ANOVA followed by Tukey's multiple comparisons test. adjusted p-values (top to bottom, left to right): p=0.0052, p=0.0021, p=0.0065. **(S4D)** Degranulation assay of Cl12 TCR-transduced T cells after 6h of co-culture with HLA-matching EBV-only or puromycin-selected EK LCLs. Frequency of CD107a⁺ out of CD8⁺ T cells normalized to the frequency of CD107a⁺ untransduced T cells. Graph shows boxplots and individual values of 2 independent experiments, with T cells from 4 different donors, tested against 2 different LCLs. Paired two-tailed t-test. **(A-C)** $^{*}p < 0.05$, $^{**}p < 0.01$, $^{***}p < 0.001$, $^{****}p < 0.0001$

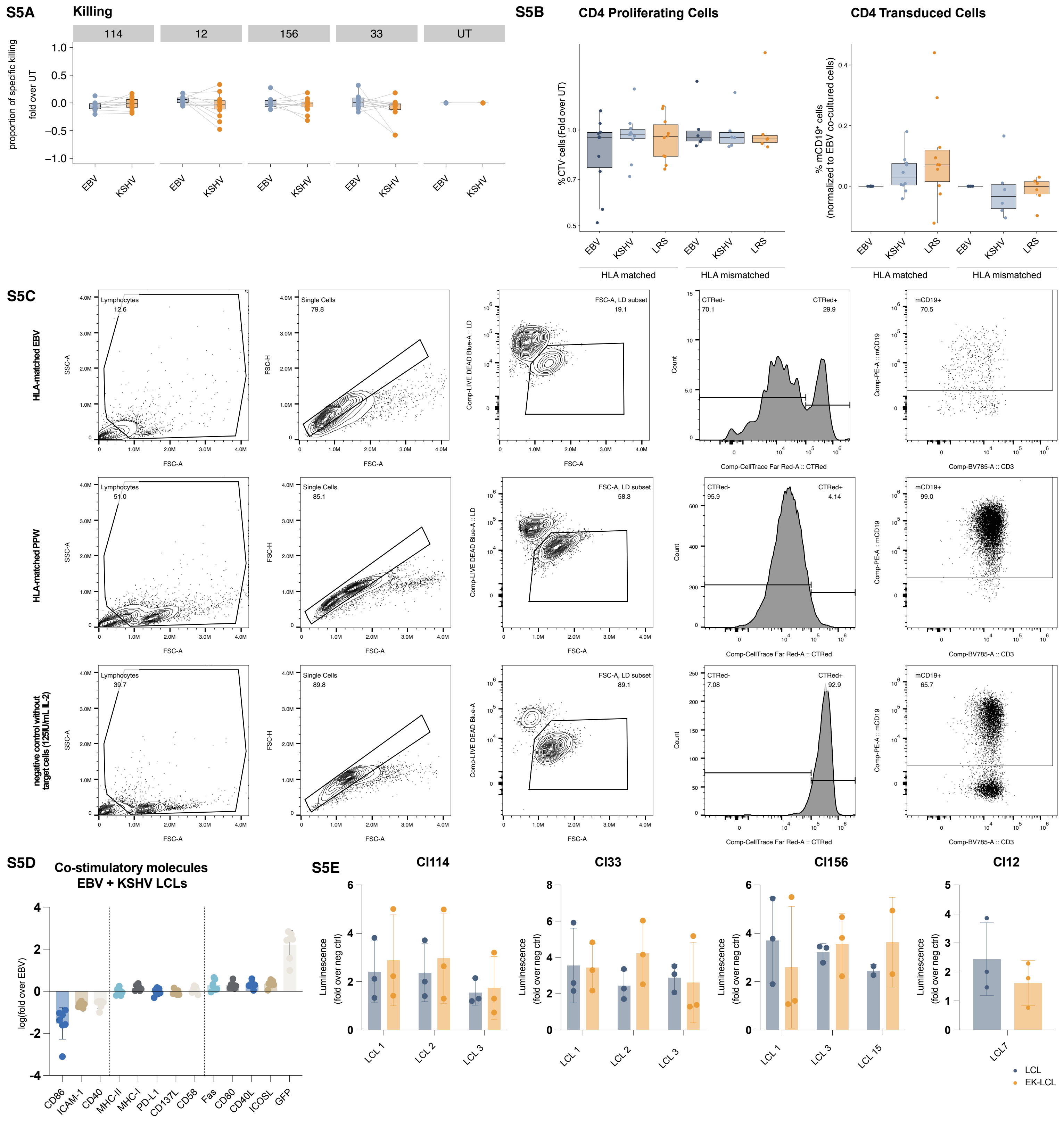

Figure S5 TCR-transduced T cells fail to recognize KSHV-infected lymphoblastoid cell lines: (S5A) Specific killing of HLA-matched EBV-only or puromycin-selected EK LCLs after co-culture with TCR-transduced T cells. Specific killing is normalized to untransduced T cells of the same donor stimulated with the identical target cells. Graph shows boxplots and individual values of 3 (Cl12, Cl33, Cl156) or 2 (Cl114) independent experiments, with T cells from 6 (Cl33, Cl156), 5 (Cl12) or 3 (Cl114) different donors, tested against 2 (Cl12) or 5 (Cl114, Cl33, Cl156) different LCLs. Paired two-tailed t-test, all p>0.05. **(S5B)** Normalized frequency of CTV-diluted or mCD19 expressing CD4$^+$ T cells after 7 days co-culture of Cl33-transduced CD4$^+$ T cells with irradiated target cells. Target cells were HLA-matched or mismatched EBV-only LCLs (EBV), puromycin-selected EK LCLs (KSHV) or peptide pulsed LCLs (LRS). Frequencies were normalized to untransduced T cells co-cultured in the same conditions. Graph shows boxplot and individual values of two independent experiments with 4 different T cell donors and 4 (HLA-matched) or 2 (HLA-mismatched) different target cell donors. Linear mixed model fit by REML taking T cell and LCL donor into account as a random effect with Tukey's post-hoc test for multiple comparisons. Reported p-values are adjusted with the Holm method. All adjusted p-values >0.05. **(S5C)** Gating strategy of the proliferation assay to quantify the frequency of proliferating and transduced cells. Single, live lymphocytes were gated for CellTrace Far Red^{low} cells and transduced mCD19$^+$ cells. The gate for CellTrace Far Red^{low} cells was set based on the negative control. **(S5D)** Geometric MFI of indicated markers on selected EK LCLs normalized to EBV only LCLs measured by flow cytometry. Graph shows mean ± SD of LCLs from n=7 different donors. **(S5E)** Jurkat Lucia NFAT reporter assay of transgenic TCR-transduced Jurkat Lucia NFAT cells stimulated with HLA-matched EBV-only or EK LCLs from different donors (LCL1-15). Graph shows the mean fold change luminescent signal normalized to the background of unstimulated cells ± SD of n=3 independent experiments. Ordinary two-way ANOVA followed by Šidák's multiple comparisons test, all p>0.05. **(A-E)** *p < 0.05, **p < 0.01, ***p < 0.001, ****p < 0.0001

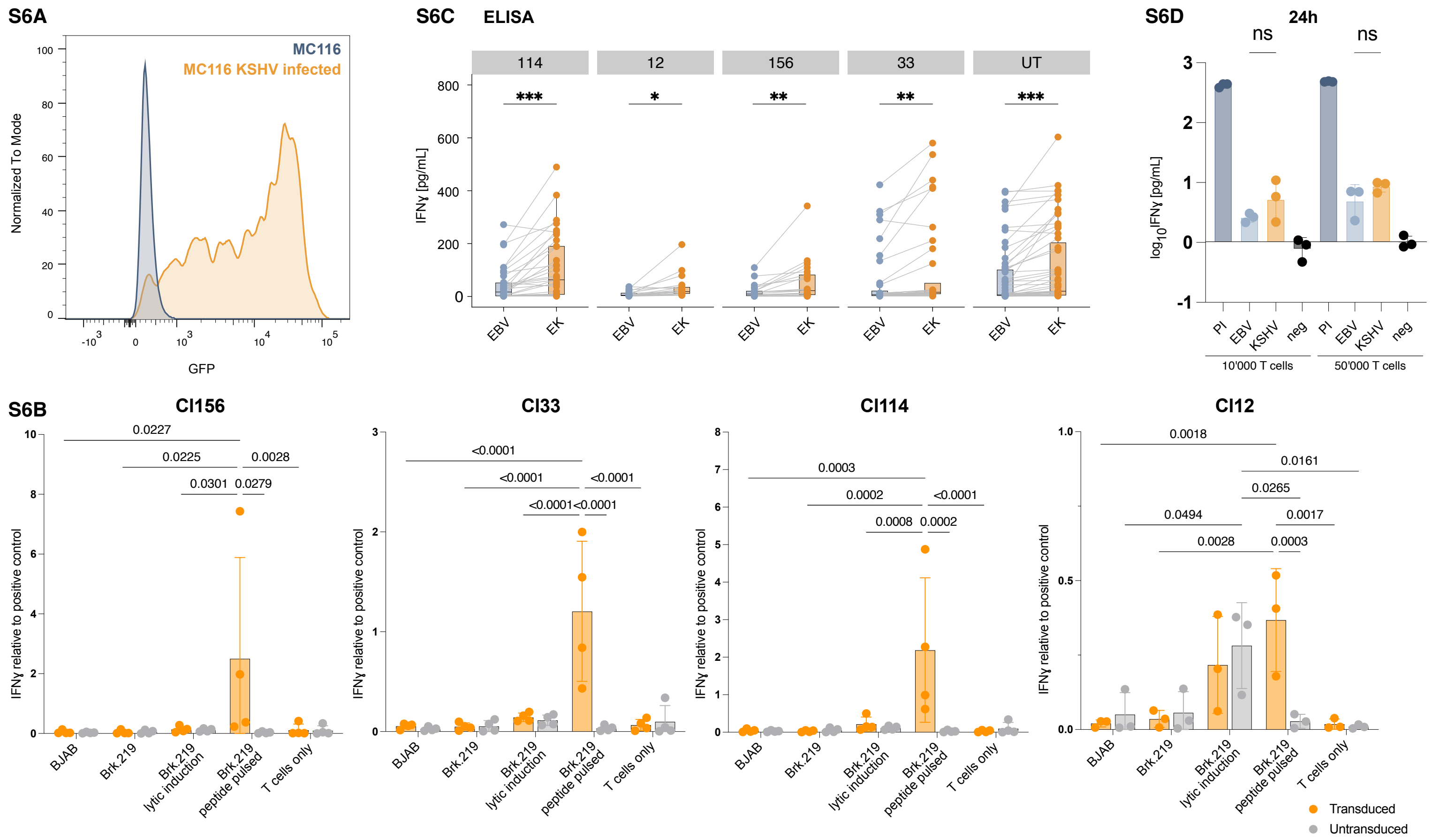

Figure S6 TCR-transduced T cells fail to recognize freshly KSHV-infected B cells: (S6A) Representative flow cytometry histogram plot of GFP expression in MC116 cells 48h post-infection or in uninfected control cells. **(S6B)** IFNɣ ELISA of TCR-transduced or untransduced T cells after overnight co-culture with cognate peptide-pulsed, lytic induced or untreated BJAB-derived Brk.219 producer cell lines, BJAB cells only or with medium only as control. IFNɣ values are plotted relative to the positive PMA/Ionomycin control. Graph shows median ± SD of 2 independent experiments with T cells from 4 (Cl12=3) different donors. Two-way ANOVA followed by Tukey's multiple comparisons test. p-values (left to right): $p=0.0002$, $p=0.0111$, $p=0.0091$, $p=0.0036$, $p=0.0002$**(S6C)** IFNɣ ELISA of TCR-transduced T cells co-cultured with EBV-only or puromycin-selected EK LCLs, which were HLA-matched. Raw, unnormalized IFNɣ measurements are plotted. Graph shows boxplots and individual values of 5 (Cl33), 4 (Cl114, Cl12) or 3 (Cl156) independent experiments, with T cells from 8 (Cl114, Cl33, Cl12) or 6 (Cl156) different donors, tested against 6 (Cl114, Cl33), 4 (Cl156) or 3 (Cl12) different LCLs. Paired two-tailed t-test. **(S6D)** IFNɣ ELISA of 10'000 or 50'000 untransduced T cells after incubation with pre-conditioned media from EBV-only (EBV) or EBV&KSHV infected B cells (KSHV) 10 days post infection, with medium only as negative control (neg) or with PMA/Ionomycin (PI) as positive control. Supernatant was analysed 24h post-incubation. Plot shows mean ± SD of one experiment with three different autologous B and T cell donor pairs. Ordinary one-way ANOVA of $\log_{10}$-transformed IFNɣ measurements followed by Tukey's multiple comparisons test. **(A-D)** *$p < 0.05$, **$p < 0.01$, ***$p < 0.001$, ****$p < 0.0001$

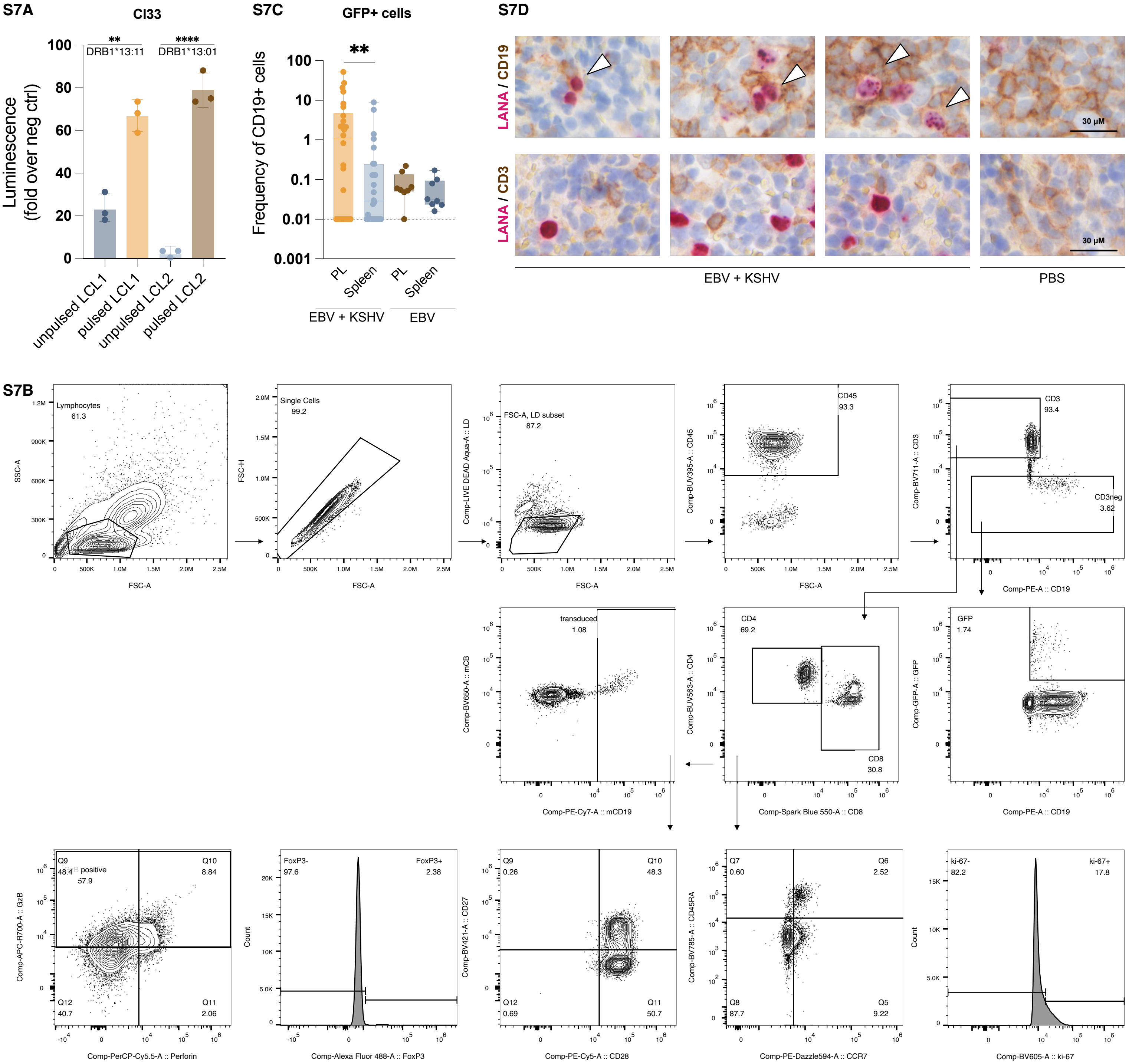

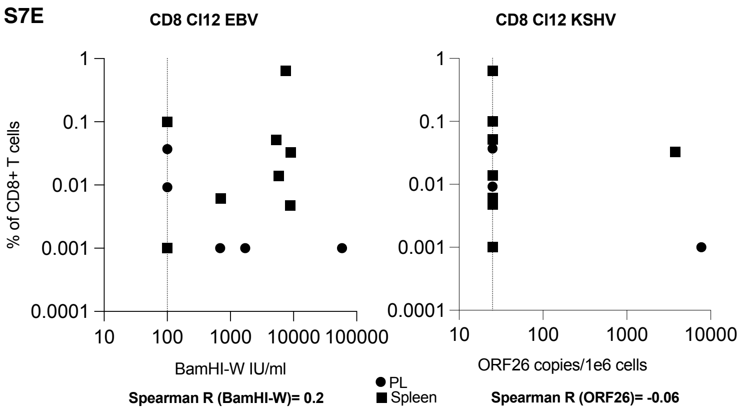

Figure S7 *In vivo* characterization of TCR-transduced T cells: (S7A) Jurkat Lucia NFAT reporter assay of Cl33 TCR-transduced Jurkat Lucia NFAT cells stimulated with cognate-peptide pulsed or unpulsed LCLs derived from the HPC donors used for reconstitution of the humanized mice. Graph shows the mean fold change luminescent signal normalized to the background of unstimulated cells ± SD of one experiment. Unpaired two-tailed t-test, p=0.0017, p<0.0001. **(S7B)** Gating strategy to assess the frequency of transduced T cells and of GFP^{+} cells. Single, live lymphocytes were gated for hCD45, hCD3 positive and negative fraction. CD3^{-} cells were gated for GFP^{+}CD19^{+} cells. CD3^{+} cells were gated for CD4^{+} or CD8^{+} cells, before gating on transduced cells based on mCD19. Transsduced or CD4 T cells were further gated for Foxp3^{+} Treg cells, CD45^{-}CCR7^{-} CD28^{+}CD27^{+} EM1 or CD28^{+}CD27^{-} EM4 cells, proliferating Ki-67^{+} and cytotoxic Granzyme B^{+} cells. **(S7C)** Frequency of GFP^{+} B cells in the peritoneal lavage (PL) and spleen of EBV & KSHV co-infected or EBV-only infected humanized mice. Graph shows boxplots and individual values of 4 (EBV & KSHV) or 2 (EBV) independent experiments with n=26 (EBV&KSHV) and n=8 (EBV) animals. Paired two-tailed t-test of $\log_{10}$-transformed values, p=0.0016. **(S7D)** Histological stainings of tissue sections (lymph node and spleen) from EBV KSHV co-infected or PBS mock infected humanized mice. LANA (nuclear fast red) was co-stained with surface CD19 (DAB) or surface CD3 (DAB). Arrows indicate examples of LANA/CD19 double-positive cells. No co-staining for CD3 and LANA was detected. **(S7E)** Correlation analysis of KSHV (ORF26) and EBV (BamHI-W) viral loads with the frequency of Cl12 transgenic out of CD8^{+} T cells. Viral loads below the detection limit were set to half of the detection limit (dashed lines). Nonparametric Spearman correlation, p=0.84 (ORF26), p=0.45 (BamHI-W). **(A-D)** *p < 0.05, **p < 0.01, ***p < 0.001, ****p < 0.0001

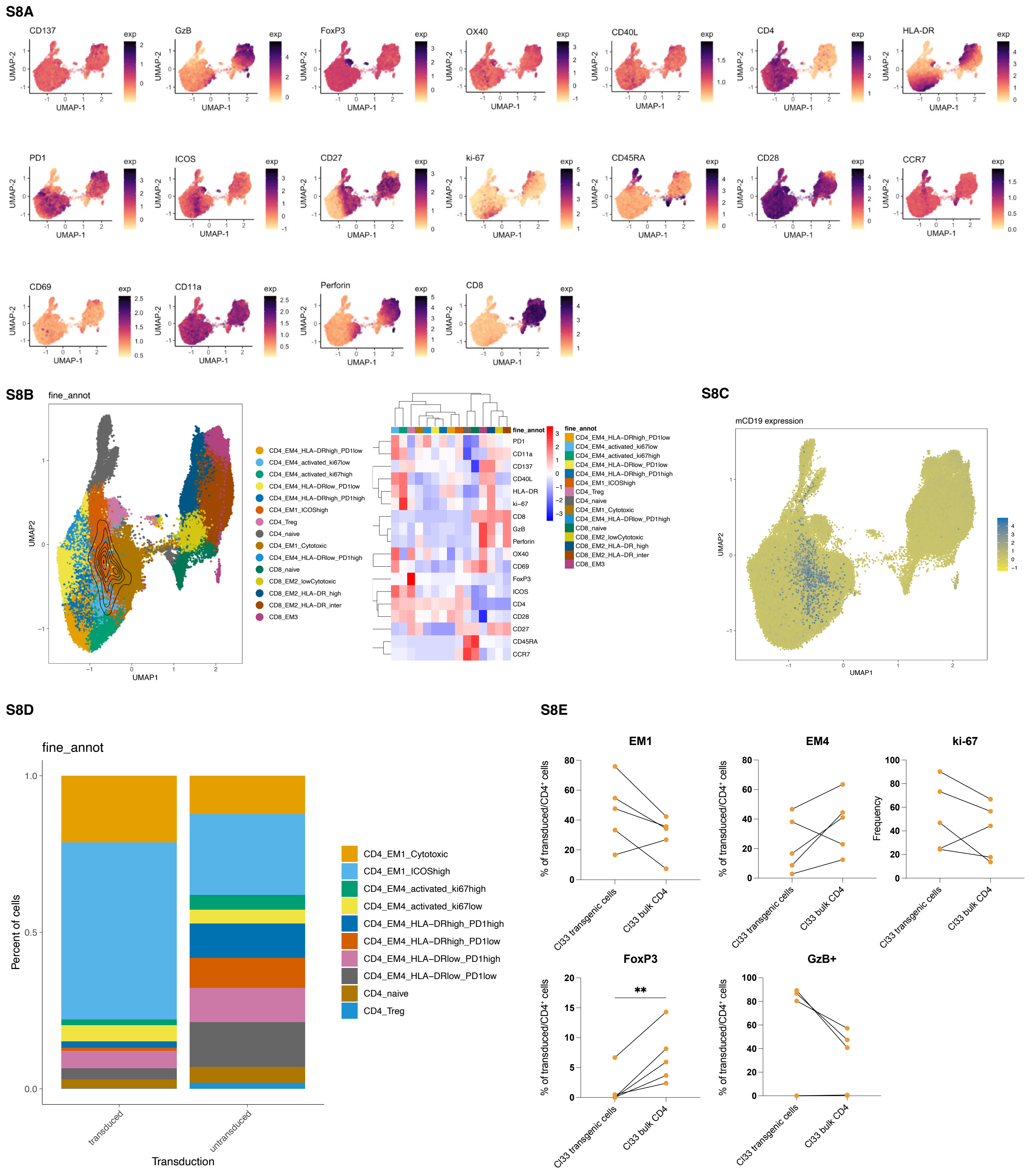

Figure S8 High dimensional analysis of TCR-transduced T cells in the peritoneum of KSHV-infected humanized mice: (S8A) Normalized expression of each marker used for clustering analysis plotted in the UMAP space. **(S8B)** UMAP visualization and high resolution FlowSOM clustering (left) and z-score normalized average expression of T cell markers per fine annotated cluster (right). mCD19 and mCB were excluded from the UMAP and clustering analysis, but mCD19$^+$ T cells are highlighted by an overlaid contour plot on the UMAP. **(S8C)** Normalized mCD19 expression in the UMAP space. **(S8D)** Stacked bar plot of frequencies of fine cluster annotations per cell within TCR-transduced T cells and untransduced CD4$^+$ T cells. **(S8E)** Frequency of cells manually gated for CD45RA$^-$CCR7$^-$CD27$^+$(EM1), CD45RA$^-$CCR7$^-$CD27$^-$(EM4), FoxP3$^+$, GzmB$^+$ or Ki-67$^+$ cells within Cl33 TCR-transduced or untransduced CD4$^+$ T cells. Graphs show individual values of 5 animals from 2 independent experiments. Two animals with <10 transduced cells were excluded from the analysis. Paired t-test, two-sided p-value, p-values (top to bottom, left to right): p=0.09, p=0.17, p=0.25, p=0.0098, p=0.09. **(A-E)** *$p < 0.05$, **$p < 0.01$, ***$p < 0.001$, ****$p < 0.0001$

SUPPLEMENTARY TABLE 1: KEY RESOURCE TABLE

Antigen	Fluorophore	Clone	Catalogue Number	Company	Dilution
anti-HHV8 LANA		LN53	ab4103	abcam	25
anti-human CD107a	FITC	H4A3	555800	BD	50
anti-human CD107a	PerCP-Cy5.5	H4A3	328616	Biolegend	50
anti-mouse TCR beta	BV650	H57-597	109251	Biolegend	25
anti-mouse TCR ß chain	B605	H57-597	109241	Biolegend	25
anti-mouse TCR ß chain	BV510	H57-597	109234	Biolegend	25
anti-Tubulin		DM1A	NB100-690	Novus	
CCR7	PE-Dazzle5994	G04H7	353236	Biolegend	100
CD11a	PerCP	TS2/4	350608	Biolegend	100
CD137	APC-Fire750	4B4-1	309834	Biolegend	100
CD137L	APC	5F4	311506	Biolegend	50
CD154	BUV496	24-31	752853	BD	50
CD244	FITC	C1.7	329506	Biolegend	100
CD25	BV711	M-A251	356138	Biolegend	100
CD27	BV421	O323	302824	Biolegend	75
CD28	PE-Cy5	CD28.2	555730	BD	10
CD34	APC	581	CD34-581-05	Invitrogen	50
CD38	PE	HIT2	303506	Biolegend	100
CD40	BV605	5C3	334336	Biolegend	50
CD40L	BV785	24-31	310842	Biolegend	50
CD45RA	BV785	HI100	304140	Biolegend	100
CD58	PE-Cy7	TS2/9	330916	Biolegend	50
CD69	Pacific Blue	FN50	310920	Biolegend	100
CD80	BV510	2D10	305234	Biolegend	50
CD86	BV785	IT2.2	305442	Biolegend	50
Fas	BV785	DX2	305646	Biolegend	50
FoxP3	FITC	259D	320106	Biolegend	100
Granzyme B	Alexa Fluor 700	QA18A28	396426	Biolegend	50
hCD19	APC	HIB19	555415	BD	100
hCD19	BUV661	HIB19	741604	BD	100
hCD19	PE	HIB19	302208	Biolegend	100
hCD19	PE-Cy7	HIB19	302216	Biolegend	100
hCD3	BV711	OKT3	317328	Biolegend	50
hCD3	PE	UCHT1	300408	Biolegend	100
hCD3	BUV395	UCHT1	563546	BD	100
hCD3	BV785	OKT3	317330	Biolegend	100
hCD4	BUV563	SK3	612912	BD	100
hCD4	PB	RPA-T4	300521	Biolegend	100
hCD4	APC-Cy7	RPA-T4	300518	Biolegend	100
hCD4	BUV496	SK3	612936	BD	50
hCD45	BUV395	HI30	563792	BD	100
hCD45	PB	HI30	304029	Biolegend	50
hCD8	BUV563	RPA-T8	612914	BD	200
hCD8	PerCP	SK1	344708	Biolegend	100
hCD8	Spark Blue 550	SK1	344760	Biolegend	100
hCD8	PE-Cy7	RPA-T8	301012	Biolegend	50
hHLA-DR	FITC	L243	307604	Biolegend	50
HLA-ABC	PE	W6/32	12-9983-42	eBiosciences	100
HLA-DR	APC-Cy7	L243	307618	Biolegend	200

HLA-DR	BUV661	G46.6	612981	BD	100
HLA-DR,DP,DQ	PE-Cy7	Tü39	361708	Biolegend	200
hNKp46	APC	9-E2	558051	BD	100
ICAM-1	BV480	HA58	746638	BD	50
ICOS	APC-Cy7	C398.4A	313530	Biolegend	50
ICOS	BUV805	DX29	748903	BD	50
ICOSL	PerCP-Cy5.5	2D3	309418	Biolegend	50
IFNɣ	APC	4S.B3	502512	Biolegend	50
ki-67	BV605	ki-67	350522	Biolegend	50
mCD19	PE	1D3/CD19	152408	Biolegend	200
mCD19	PE-Cy7	1D3/CD19	152418	Biolegend	200
OX40	Alexa Fluor 647	Ber-ACT35	350018	Biolegend	50
OX40	PE-Cy7	Ber-ACT35	350012	Biolegend	50
PD-1	BUV737	EH12.1	612792	BD	50
PD-L1	APC	29E.2A3	329708	Biolegend	50
Perforin	PerCP-Cy5.5	δG9	563762	BD	50
peroxidase-conjugated goat anti-rat		polyclonal	112-035-003	Jackson ImunoResearch	
TNF	PE	MAB11	502909	Biolegend	50
Vß1	PE	BL37.2	IM2355	Beckman Coulter	30
Vß11	FITC	C21	IM1586	Beckman Coulter	30
Vß12	PE	VER2.32.1	IM2291	Beckman Coulter	30
Vß13.1	PE	IMMU 222	IM2292	Beckman Coulter	30
Vß13.6	FITC	JU74.3	IM1330	Beckman Coulter	30
Vß14	PE	CAS1.1.3	IM2047	Beckman Coulter	30
Vß16	FITC	TAMAYA1.2	IM1560	Beckman Coulter	30
Vß17	FITC	E17.5F3.15.13	IM1234	Beckman Coulter	30
Vß18	PE	BA62.6	IM2049	Beckman Coulter	30
Vß2	PE	MPB2D5	IM2213	Beckman Coulter	30
Vß20	PE	ELL1.4	IM2295	Beckman Coulter	30
Vß21.3	FITC	IG125	IM1483	Beckman Coulter	30
Vß22	FITC	IMMU 546	IM1483	Beckman Coulter	30
Vß23	PE	AF23	IM2004	Beckman Coulter	30
Vß3	FITC	CH92	IM2372	Beckman Coulter	30
Vß4	PE	CAS1.1.3	IM2047	Beckman Coulter	30
Vß5.1	FITC	IMMU157	IM1552	Beckman Coulter	30
Vß5.2	FITC	36213	IM1482	Beckman Coulter	30
Vß5.3	PE	3D11	IM2002	Beckman Coulter	30
Vß7.1	PE	ZOE	IM2287	Beckman Coulter	30
Vß8	FITC	56C5.2	IM1233	Beckman Coulter	30
Vß9	PE	FIN9	IM2003	Beckman Coulter	30

**Reagent**	**Source**	**Stock number**
0.25% Trypsin-EDTA	Gibco	7001619
7AAD	Invitrogen	A1310
Agel-HF	NEB	R3552S
anti-human IgM u-specific chain, southern biotech	BioConcept	2020-01
Bovine Serum Albumin Fraction IV	Carl Roth	8076.1
Brefeldin A	Sigma-Aldrich	B5936-200UL
BSRG1-HF	NEB	R3575L
DMEM	Life Technologies	7001566
DPBS	Gibco	10010031
EDTA Lösung pH 8.0 (0.5M) für die Molekularbiologie	NeoFroxx	1353ML500
Ethanol	VWR	64-17-5
Fc Receptor Blocking Reagent	Miltenyi	130-059-901
Fetal bovine serum	Merck	S0615-500ML
Ficoll Paque	Merck	17-5442-03
hrIL-15	Biolegend	570304
hrIL-2	Preprotech	200-02
hrIL-7	Miltenyi Biotec	130-095-362
Human AB serum	BioConcept	2-13F06-H
Hygromycin	Invitrogen	7001580
IMDM	Gibco	21980032
Ionomycin	Sigma	I3909
$KHCO_3$	Carl Roth	9437.2
L-Glutamine	Thermofisher	25030081
NH_4CL	Carl Roth	K298.1
NotI	NEB	R0189L
OptiMEM	life technology	31985-047
p8.91 plasmid	Addgene	#187441
PEG-it	BioCat	LV825A-1
PEI MAX	Polysciences Inc.	24765-100
PEI MAX ®	Polysciences Inc.	24765-100
Penecillin-Streptomycin	Thermofisher	15140122
Peptides	Peptides & elephants	
phorbol-12-myristat-13-acetat (PMA)	Sigma	P1585-1MG
Polybrene	Merek	TR-1003-G
polyvinylidene difluoride membrane	VWR	10600023
Puromycin	Sigma	P7255-25MG
QUANTI-LucTM 4 Lucia/Guassia	InvivoGen	rep-qlc4lg5
RPMI-1640	Gibco	7001612
Sodium butyrate	Merck	303410
Super Bright Complete Staining Buffer	eBioscience	SB-4401-42
TaqMan™ Universal PCR Master Mix	Applied Biosystems	4305719
Tween20	Carl Roth	9127.1
VSV.G	Addgene	#14888
Zeocin	InvivoGen	ant-zn-1

Kit name	Source	Stock number
CD3/CD28 Dynabeads	Thermo Fisher/Gibco	11131D
CellTraceTM Far Red Cell Proliferation kit	Invitrogen	C34564
Chromium Next GEM Single Cell 5’ Reagent Kits v2 (Dual Index)	10x Genomics	
Cytofix/Cytoperm Fixation/Permeabilization Kit	BD Biosciences	554655
Direct CD34 Progenitor Cell Isolation Kit	Miltenyi	130-046-703
DNeasy Blood & Tissue Kit	Qiagen	7002196
ELISA flex: Human IFNɣ (HRP)	Mabtech	3420-1H
ELISA flex: Human TNFa (HRP)	Mabtech	3512-1H
Foxp3 / Transcription Factor Staining Buffer Set	Thermofisher	00-5523-00
Gibson Assembly® Cloning Kit	NEB	E5510S
Human CD19 MicroBeads	Miltenyi	130-050-301
Human CD4 MicroBeads	Miltenyi	130-097-048
Human CD4 T cell isolation kit	Miltenyi	130-096-533
Human CD8 Microbeads	Miltenyi	130-045-201
Human CD8 T cell isolation kit	Miltenyi	130-096-495
LIVE/DEAD™ Fixable Blue Dead Cell Stain Kit, for UV excitation	Invitrogen	L23105
MycoSpy Mastermix	Biontex	M020-050
red PKH membrane label kit	Sigma-Aldrich	MINI26-1KT
RNeasy Mini kit	Qiagen	74106
TaqMan® Universal PCR Master Mix	Applied Biosystems	7001524
WesternBright Sirius HRP kit	advansta	K-12043-D20
Zombie Aqua Fixable Viability Kit	Biolegend	423101
Zombie NIR™ Fixable Viability Kit	Biolegend	423105

Primer Name	Sequence	Purpose	Company
BAMHI W fw	CTTCTCAGTCCAGCGCGTTT	qPCR	microsynth
BAMHI W rev	CAGTGGTCCCCCTCCCTAGA	qPCR	microsynth
BAMHI W Probe	CGTAAGCCAGACAGCAGCCAATTGTCAG	qPCR	microsynth
GAPDH fw	CAAGGTCATCCATGACAACTTTG	qPCR	microsynth
GAPDH rev	GGCCATCCACAGTCTTCTGG	qPCR	microsynth
GAPDH Probe	ACCACAGTCCATGCCATCACTGCCA	qPCR	microsynth
ORF26 fw	GCTCGAATCCAACGGATTTG	qPCR	microsynth
ORF26 rev	AATAGCGTGCCCCAGTTGC	qPCR	microsynth
ORF26 probe	TTCCCCATGGTCGTGCCTC	qPCR	microsynth

**Machine/Software**	**Company**
Aria III 5L	BD Biosciences
Aurora 5L spectral flow cytometer	Cytek
CFX384 Touch Real-Time PCR Detection System	bioRad
Decombinator software v4.2	https://github.com/innate2adaptive/Decombinator
DxH500 Hematology Analyzer	Beckman Coulter
FACSCanto II	BD Biosciences
FlowJoTM Software	v10, BD
Fusion FX	Vilber Smart Imaging
GraphPhad Prism	GraphPad Software, Boston, Massachusetts USA
Image J / Fiji	
Infinite 200 PRO	Tecan
Loupe VDJ Browser	Cell Ranger, 10X genomics
LSRFortessa	BD Biosciences
MiSeq	Illumina
Novaseq 6000	Illumina
NucliSENS easyMag	bioMérieux
Posit team (2025). RStudio: Integrated Development Environment for R.	Posit Software, PBC, Boston, MA.
S6 5L	BD Biosciences

Experimental models: Cell lines	Source	Stock number
Raji Cells		
HEK293T Cells		
Jurkat-LuciaTM NFAT reporter cells	InvivoGen	jktl-nfat
PBMCs	Blutspende SRK Zürich	
T2B35 Cells	Rajiv Khanna, Brisbane, Australia	
VG-1 PEL Cell line	Alexander Hahn, Göttingen, Germany	
MC116 cells	Alexander Hahn, Göttingen, Germany	

**Microorganism**	**Source**	**Stock number**
Recombinant EBV B95-8-GFP (EBVwt)	Kalla et al., 2012	NA
EBV B95-8 (EBVwt)	Miller et al, PNAS, 1972	NA
Recombinant KSHV-GFP (rKSHV.219)	Kati et al., 2015	NA
iSLK.219	Brulois et al, 2012	NA

Experimental models: Mouse strains	Source	Stock number
NOD.Cg-Prkdcscid Il2rgtm1Wjl/SzJ (NSG)	The Jackson Laboratory	005557
