# Supplementary material for: LANA-specific CD4+ effector T cells accumulate at the site of KSHV infection in humanized mice
Source: Nat Commun. 2025 Dec 5;17:282. doi: 10.1038/s41467-025-66992-2 (PMC12783722; doi:10.1038/s41467-025-66992-2)
Supplement: Supplementary file 2 — Reporting Summary [file 41467_2025_66992_MOESM2_ESM.pdf]

nature portfolio

Corresponding author(s): Christian Münz

Last updated by author(s): Nov 19, 2025

Reporting Summary

Nature Portfolio wishes to improve the reproducibility of the work that we publish. This form provides structure for consistency and transparency in reporting. For further information on Nature Portfolio policies, see our Editorial Policies and the Editorial Policy Checklist.

Statistics

For all statistical analyses, confirm that the following items are present in the figure legend, table legend, main text, or Methods section.

n/a	Confirmed	
☐	☒	The exact sample size (n) for each experimental group/condition, given as a discrete number and unit of measurement
☐	☒	A statement on whether measurements were taken from distinct samples or whether the same sample was measured repeatedly
☒	☐	The statistical test(s) used AND whether they are one- or two-sided<br>*Only common tests should be described solely by name; describe more complex techniques in the Methods section.*
☐	☒	A description of all covariates tested
☐	☒	A description of any assumptions or corrections, such as tests of normality and adjustment for multiple comparisons
☒	☐	A full description of the statistical parameters including central tendency (e.g. means) or other basic estimates (e.g. regression coefficient) AND variation (e.g. standard deviation) or associated estimates of uncertainty (e.g. confidence intervals)
☐	☒	For null hypothesis testing, the test statistic (e.g. F, t, r) with confidence intervals, effect sizes, degrees of freedom and P value noted<br>*Give P values as exact values whenever suitable.*
☒	☐	For Bayesian analysis, information on the choice of priors and Markov chain Monte Carlo settings
☒	☐	For hierarchical and complex designs, identification of the appropriate level for tests and full reporting of outcomes
☒	☐	Estimates of effect sizes (e.g. Cohen's d, Pearson's r), indicating how they were calculated

Our web collection on statistics for biologists contains articles on many of the points above.

Software and code

Policy information about availability of computer code

Data collection	SpectroFlo Software was used for QC, sample acquisition and spectral unmixing or BD FACSDiva Software was used for sample acquisition and compensation of flow cytometry Data. CFX Manager Software was used to collect rt-qPCR data.
Data analysis	SpectroFlo v3.0, FlowJoTM Software (v10, BD), GraphPad Prism 10, R Statistical Software (v4.2.0; R Core Team 2021), imageJ/Fiji, Phenochart (v1.0) and InForm (v2.4.8) software, Cyclone pipeline (Patel et al., 2023), R packages (lme4, multcomp, dittoSeq, SingleCellExperiment, Cyclone, BiocStyle, tidyverse, dplyr, ggplot2), CLC Main Workbench 22.The Decombinator software v4.2 available on Github (https://github.com/innate2adaptive/Decombinator) and Loupe VDJ Browser (Cell Ranger, 10X genomics) was used to analyze the TCR sequencing data.

For manuscripts utilizing custom algorithms or software that are central to the research but not yet described in published literature, software must be made available to editors and reviewers. We strongly encourage code deposition in a community repository (e.g. GitHub). See the Nature Portfolio guidelines for submitting code & software for further information.

Data

Policy information about availability of data

All manuscripts must include a data availability statement. This statement should provide the following information, where applicable:

- Accession codes, unique identifiers, or web links for publicly available datasets
- A description of any restrictions on data availability
- For clinical datasets or third party data, please ensure that the statement adheres to our policy

The raw TCR-sequencing data generated in this study have been deposited in the European Nucleotide Archive (ENA) under accession code PRJEB101731 (https://www.ebi.ac.uk/ena/browser/view/PRJEB101731) for bulk TCR sequencing and under accession code PRJEB103950 (https://www.ebi.ac.uk/ena/browser/view/PRJEB103950) for VDJ scRNAseq data. The processed TCR sequences corresponding to confirmed functional KSHV-specific TCRs are provided in the Source Data file. Processed data supporting the findings of this study, including the consensus annotations for Cl114 are provided in the source data file.

Research involving human participants, their data, or biological material

Policy information about studies with human participants or human data. See also policy information about sex, gender (identity/presentation), and sexual orientation and race, ethnicity and racism.

Reporting on sex and gender	Sex information of anonymous T cell and B cell donors used for in vitro experiments are not available. Experiments were not designed to find a sex-effect.
Reporting on race, ethnicity, or other socially relevant groupings	*Please specify the socially constructed or socially relevant categorization variable(s) used in your manuscript and explain why they were used. Please note that such variables should not be used as proxies for other socially constructed/relevant variables (for example, race or ethnicity should not be used as a proxy for socioeconomic status).* *Provide clear definitions of the relevant terms used, how they were provided (by the participants/respondents, the researchers, or third parties), and the method(s) used to classify people into the different categories (e.g. self-report, census or administrative data, social media data, etc.)* *Please provide details about how you controlled for confounding variables in your analyses.*
Population characteristics	All blood donors fulfilled the admission criteria for blood donations of the Swiss Transfusion Swiss Red Cross Zurich. No data was available about the donors (e.g. sex, age).
Recruitment	All donors voluntarily donated blood to the Swiss Transfusion Swiss Red Cross Zurich. There was no recruitment process involved from our site.
Ethics oversight	The use of peripheral blood samples from healthy donors as well as the use of human fetal liver tissue was authorized by the cantonal ethical committee of Zurich, Switzerland (KEK-ZH-NR. 2019-00837).

Note that full information on the approval of the study protocol must also be provided in the manuscript.

Field-specific reporting

Please select the one below that is the best fit for your research. If you are not sure, read the appropriate sections before making your selection.

☒ Life sciences ☐ Behavioural & social sciences ☐ Ecological, evolutionary & environmental sciences

For a reference copy of the document with all sections, see nature.com/documents/nr-reporting-summary-flat.pdf

Life sciences study design

All studies must disclose on these points even when the disclosure is negative.

Sample size	No sample size calculation was performed for in vitro experiments, however, whenever enough HLA-matching donors were available, three different human donors were used at least. For animal experiments, the primary statistical strategy was descriptive statistics with inferential statistics as secondary strategy, taking into account the exploratory nature of the experiments and absence of prior power analysis for sample size determination and hypothesis testing. However, prior exemplary calculations to evaluate the necessary number of observations n to detect an effect size of f=0.5 with a power of 0.8 and a Bonferroni-corrected significance level of 0.05 was calculated for the continuous outcome of "immune cell numbers" by means of an F test using R Statistical Software (R Core Team 2021) with webPower package (Zhang 2023), and for the categorical outcome "proportion of KSHV-infected animals" by means of Chi-square test for two proportions with R Statistical Software (R Core Team 2021) with pwr package (Champely 2020). The calculated sample size for the categorical outcome "proportion of KSHV-infected animals" was not met with the two conducted animal experiments.
Data exclusions	For Figure 5 D-G, two animals with less than 10 transgenic cells were excluded from the analysis. These animals had no detectable KSHV viral loads during the whole course of the experiment.
Replication	In vitro experiments were performed with cells from different human donors and on different days according to the same standardized protocols to ensure reproducibility. Replicates are indicated in the figure legends. Animal experiments were repeated twice with hematopoietic stem cells of different donors. All findings were reproduced in the second experiment, except the cytotoxic CD4 T cell differentiation, which might be explained by donor-specific effects.

Randomization	We employed stratified randomization for the allocation of the treatment groups to the experimental units (the animals) from the same human stem cells derived within the same donor. The donors are assumed to be independent. The stratification is to ensure homogeneity based on percentage of reconstitution, of biological requirements of the human donors and based on sex as balanced as possible but not to find sex-effect.
Blinding	Blinding was not feasible for most experiments. As is typical for basic research involving cell culture and animal models, the researchers responsible for data acquisition were necessarily involved in experimental design and execution. Data analysis of flow cytometry data for figure 5 was carried out in an unbiased manner. Histological stainings and immunoblotting were performed in a blinded manner. No additional blinding procedures were applied.

Reporting for specific materials, systems and methods

We require information from authors about some types of materials, experimental systems and methods used in many studies. Here, indicate whether each material, system or method listed is relevant to your study. If you are not sure if a list item applies to your research, read the appropriate section before selecting a response.

Materials & experimental systems

n/a	Involved in the study
☐	☒ Antibodies
☐	☒ Eukaryotic cell lines
☒	☐ Palaeontology and archaeology
☐	☒ Animals and other organisms
☒	☐ Clinical data
☒	☐ Dual use research of concern
☒	☐ Plants

Methods

n/a	Involved in the study
☒	☐ ChIP-seq
☐	☒ Flow cytometry
☒	☐ MRI-based neuroimaging

Antibodies

Antibodies used

See also methods section and key resource table in supplement file:
Vß11 FITC C21 IM1586 Beckman Coulter 30
Vß12 PE VER2.32.1 IM2291 Beckman Coulter 30
Vß13.1 PE IMMU 222 IM2292 Beckman Coulter 30
Vß13.6 FITC JU74.3 IM1330 Beckman Coulter 30
Vß14 PE CAS1.1.3 IM2047 Beckman Coulter 30
Vß16 FITC TAMAYA1.2 IM1560 Beckman Coulter 30
Vß17 FITC E17.5F3.15.13 IM1234 Beckman Coulter 30
Vß18 PE BA62.6 IM2049 Beckman Coulter 30
Vß2 PE MPB2D5 IM2213 Beckman Coulter 30
Vß20 PE ELL1.4 IM2295 Beckman Coulter 30
Vß21.3 FITC IG125 IM1483 Beckman Coulter 30
Vß22 FITC IMMU 546 IM1483 Beckman Coulter 30
Vß23 PE AF23 IM2004 Beckman Coulter 30
Vß4 PE CAS1.1.3 IM2047 Beckman Coulter 30
Vß5.1 FITC IMMU157 IM1552 Beckman Coulter 30
Vß5.2 FITC 36213 IM1482 Beckman Coulter 30
Vß8 FITC 56C5.2 IM1233 Beckman Coulter 30
Vß5.3 PE 3D11 IM2002 Beckman Coulter 30
Vß7.1 PE ZOE IM2287 Beckman Coulter 30
Vß9 PE FIN9 IM2003 Beckman Coulter 30
anti-HHV8 LANA LN53 ab4103 abcam 25
anti-human CD107a FITC H4A3 555800 BD 50
anti-human CD107a PerCP-Cy5.5 H4A3 328616 Biolegend 50
anti-mouse TCR beta BV650 H57-597 109251 Biolegend 25
anti-mouse TCR ß chain B605 H57-597 109241 Biolegend 25
anti-mouse TCR ß chain BV510 H57-597 109234 Biolegend 25
anti-Tubulin DM1A NB100-690 Novus
CCR7 PE-Dazzle5994 G04H7 353236 Biolegend 100
CD11a PerCP TS2/4 350608 Biolegend 100
CD137 APC-Fire750 4B4-1 309834 Biolegend 100
CD137L APC 5F4 311506 Biolegend 50
CD154 BUV496 24-31 752853 BD 50
CD244 FITC C1.7 329506 Biolegend 100
CD25 BV711 M-A251 356138 Biolegend 100
CD27 BV421 O323 302824 Biolegend 75
CD28 PE-Cy5 CD28.2 555730 BD 10

CD34 APC 581 CD34-581-05 Invitrogen 50
CD38 PE HIT2 303506 Biolegend 100
CD40 BV605 5C3 334336 Biolegend 50
CD40L BV785 24-31 310842 Biolegend 50
CD45RA BV785 HI100 304140 Biolegend 100
CD58 PE-Cy7 TS2/9 330916 Biolegend 50
CD69 Pacific Blue FN50 310920 Biolegend 100
CD80 BV510 2D10 305234 Biolegend 50
CD86 BV785 IT2.2 305442 Biolegend 50
Fas BV785 DX2 305646 Biolegend 50
FoxP3 FITC 259D 320106 Biolegend 100
Granzyme B Alexa Fluor 700 QA18A28 396426 Biolegend 50
hCD19 APC HIB19 555415 BD 100
hCD19 BUV661 HIB19 741604 BD 100
hCD19 PE HIB19 302208 Biolegend 100
hCD19 PE-Cy7 HIB19 302216 Biolegend 100
hCD3 BV711 OKT3 317328 Biolegend 50
hCD3 PE UCHT1 300408 Biolegend 100
hCD3 BUV395 UCHT1 563546 BD 100
hCD3 BV785 OKT3 317330 Biolegend 100
hCD4 BUV563 SK3 612912 BD 100
hCD4 PB RPA-T4 300521 Biolegend 100
hCD4 APC-Cy7 RPA-T4 300518 Biolegend 100
hCD4 BUV496 SK3 612936 BD 50
hCD45 BUV395 HI30 563792 BD 100
hCD45 PB HI30 304029 Biolegend 50
hCD8 BUV563 RPA-T8 612914 BD 200
hCD8 PerCP SK1 344708 Biolegend 100
hCD8 Spark Blue 550 SK1 344760 Biolegend 100
hCD8 PE-Cy7 RPA-T8 301012 Biolegend 50
hHLA-DR FITC L243 307604 Biolegend 50
HLA-DR APC-Cy7 L243 307618 Biolegend 200
HLA-DR BUV661 G46.6 612981 BD 100
hNKp46 APC 9-E2 558051 BD 100
ICAM-1 BV480 HA58 746638 BD 50
ICOS APC-Cy7 C398.4A 313530 Biolegend 50
ICOS BUV805 DX29 748903 BD 50
ICOSL PerCP-Cy5.5 2D3 309418 Biolegend 50
IFNɣ APC 4S.B3 502512 Biolegend 50
ki-67 BV605 ki-67 350522 Biolegend 50
mCD19 PE 1D3/CD19 152408 Biolegend 200
mCD19 PE-Cy7 1D3/CD19 152418 Biolegend 200
HLA-ABC PE W6/32 12-9983-42 eBiosciences 100
HLA-DR,DP,DQ PE-Cy7 Tü39 361708 Biolegend 200
OX40 Alexa Fluor 647 Ber-ACT35 350018 Biolegend 50
OX40 PE-Cy7 Ber-ACT35 350012 Biolegend 50
PD-1 BUV737 EH12.1 612792 BD 50
PD-L1 APC 29E.2A3 329708 Biolegend 50
Perforin PerCP-Cy5.5 δG9 563762 BD 50
peroxidase-conjugated goat anti-rat polyclonal 112-035-003 Jackson ImunoResearch
TNF PE MAB11 502909 Biolegend 50
Vß1 PE BL37.2 IM2355 Beckman Coulter 30
Vß3 FITC CH92 IM2372 Beckman Coulter 30

Validation

All antibodies were validated by the providers indicated above. Antibodies were further titrated as well as tested by means of FMOs and biological positive and negative controls. Panel tests were carried out before the experiments.

Eukaryotic cell lines

Policy information about cell lines and Sex and Gender in Research

Cell line source(s)

Brk.219 (Kati et al 2013, Prof. T. Schulz), Raji cells, HEK293T, B95-8 cells, HEK (p2089, Dr. W. Hammerschmidt), T2B35 cells (Dr. R. Khanna), MC116 (Dr. A. Hahn), Jurkat-Lucia NFAT reporter cells (InvivoGen), iSLK.219 (D. Ganem),

Authentication

None of the cell lines used were authenticated, except Raji cells which were confirmed by HLA-typing.

Mycoplasma contamination

Cell lines were tested on monthly basis using the MycoSpy Mastermix (Biontex, M020-050). All cell lines used for experiments were negative for mycoplasma contamination.

Commonly misidentified lines (See ICLAC register)

iSLK cells derived from SLK cells are used as KSHV virus producer cell lines, irrespective of their origin.

Animals and other research organisms

Policy information about studies involving animals; ARRIVE guidelines recommended for reporting animal research, and Sex and Gender in Research

Laboratory animals

NOD.Cg-Prkdcscid Il2rgtm1Wjl/SzJ (NSG) obtained from the Jackson Laboratories and were housed in the Laboratory Animal Services Center of the University of Zurich. Animals were injected with hematopoietic progenitor cells within the first 1-5 days after birth and 3-4 months old reconstituted animals were used for the experiments.
Animals were housed in IVC type 2 long cages (T2L) with up to 5 animals per cage. Standard enrichment was provided (bedding, red mouse house, tissues and crinklets). Temperature was maintained between 21-24°C, humidity between 40-60%. Housings had a regular 12-hour dark/12-hour light cycle.

Wild animals

The study did not involve wild animals.

Reporting on sex

Animals were reconstituted in the first 1-5 days after birth when sex was not known. Available animals were then allocated with three months of age to the experimental groups based on stratified randomization. The stratification ensured homogeneity based on percentage of reconstitution of human immune system components and on sex as balanced as possible. However, the experiment was not designed to find a sex-effect. A total of 25 female mice and 31 male mice were used. 19 female and 19 male mice were equally allocated to the experimental groups, while 6 female and 12 male mice were used as T cell donor animals. T cells from all donor animals reconstituted with the same hematopoietic progenitor cells were mixed prior to transduction and re-injection into the recipient animals. Sex disaggregated data could be provided, but the sample size was not designed to see sex-specific effects.

Field-collected samples

The study did not involve field collected samples.

Ethics oversight

The animal experiments performed were approved by the veterinary office of the canton of Zurich (KVET), Switzerland, in the licenses ZH212/2020 and ZH192/2023.

Note that full information on the approval of the study protocol must also be provided in the manuscript.

Plants

Seed stocks

Report on the source of all seed stocks or other plant material used. If applicable, state the seed stock centre and catalogue number. If plant specimens were collected from the field, describe the collection location, date and sampling procedures.

Novel plant genotypes

Describe the methods by which all novel plant genotypes were produced. This includes those generated by transgenic approaches, gene editing, chemical/radiation-based mutagenesis and hybridization. For transgenic lines, describe the transformation method, the number of independent lines analyzed and the generation upon which experiments were performed. For gene-edited lines, describe the editor used, the endogenous sequence targeted for editing, the targeting guide RNA sequence (if applicable) and how the editor was applied.

Authentication

Describe any authentication procedures for each seed stock used or novel genotype generated. Describe any experiments used to assess the effect of a mutation and, where applicable, how potential secondary effects (e.g. second site T-DNA insertions, mosiacism, off-target gene editing) were examined.

Flow Cytometry

Plots

Confirm that:

- [x] The axis labels state the marker and fluorochrome used (e.g. CD4-FITC).
- [x] The axis scales are clearly visible. Include numbers along axes only for bottom left plot of group (a 'group' is an analysis of identical markers).
- [ ] All plots are contour plots with outliers or pseudocolor plots.
- [x] A numerical value for number of cells or percentage (with statistics) is provided.

Methodology

Sample preparation

Splenocytes from mashed spleens were isolated by density gradient centrifugation at 1100g for 25 min at room temperature using Ficoll-Paque (GE Healthcare). Bone marrow cells were isolated from tibia and femur by centrifugation of cut open bones giving access to the bone marrow. The bone marrow pellet was collected in 1.5mL Eppendorf tubes and strained through a 70m filter. Blood for PBMC isolation was gained from heart puncture or weekly tail vein nicking. Blood was collected in EDTA tubes (BD Microtainer K2-EDTA tubes) and erythrocyte lysis was performed using an inhouse ACK lysis buffer. Cells from the peritoneal cavity were collected by injecting 5ml PBS containing 3% FBS, followed by aspiration of the lavage fluid. All counts of cells isolated from tissues as well as whole blood cell counts were assessed using a DxH500 Hematology Analyzer (Beckman Coulter) and aliquoted for subsequent analysis. For flow cytometry, 5x10e5 – 5x10e6 cells were used for staining. Super Bright Complete Staining Buffer (eBioscience) was included in the master mix in order to prevent polymer interactions. Single cell suspensions were stained during 30min at 4°C for surface antigens and live-dead marker in a 96-well V-bottom plate, followed by fixation and permeabilization with the Foxp3 staining kit (eBioscience)

	according to the manufacturer's instructions. For the fixation and permeabilization in the degranulation assay with intracellular cytokine staining, the Cytofix/Cytoperm Fixation/Permeabilization Kit (BD Biosciences) was used instead
Instrument	BD FACS Canto II, BD LSR II Fortessa, Cytek Aurora 5L spectral flow cytometer, BD Aria III 5L, BD S6 5L
Software	SpectroFlo Software was used for QC, sample acquisition and spectral unmixing or BD FACSDiva Software was used for sample acquisition, compensation and sorting.
Cell population abundance	TCR-expressing Jurkat Lucia NFAT reporter cells were enriched to >87.5% by sorting for live, single, murine CD19+, and murine TCR ß+ cells as determined by flow cytometric reanalysis of the sorted fraction.
Gating strategy	Cells were always gated for lymphocytes (FSC vs SSC, FSC-A threshold of 50), single cells (FSC-A vs FSC-H) and live cells (Zombie negative) before applying any other gating strategy.

☒ Tick this box to confirm that a figure exemplifying the gating strategy is provided in the Supplementary Information.
